# 6 GHz lamb wave acoustic filters based on A1-mode lithium niobate thin film resonators with checker-shaped electrodes

**DOI:** 10.1038/s41378-024-00776-4

**Published:** 2024-09-19

**Authors:** Xin Tong, Yang Zou, Zhiwei Wen, Zesheng Liu, Tiancheng Luo, Jie Zhou, Huajun Liu, Yuqi Ren, Qinwen Xu, Wenjuan Liu, Yan Liu, Yao Cai, Chengliang Sun

**Affiliations:** 1 The Institute of Technological Sciences, Hubei Key Laboratory of Electronic Manufacturing and Packaging Integration, Wuhan University, Wuhan, China; 2 Institute of Materials Research and Engineering (IMRE), Agency for Science, Technology and Research (A*STAR), Singapore, Singapore; 3 Hubei Yangtze Memory Laboratories, Wuhan, China; 4 School of Microelectronics, Wuhan University, Wuhan, China

**Keywords:** Nanoscience and technology, Electronic devices

## Abstract

The first-order antisymmetric (A1) mode lamb wave resonator (LWR) based on Z-cut LiNbO_3_ thin films has attracted significant attention and is widely believed to be a candidate for next-generation reconfigurable filters with high frequency and large bandwidth (*BW*). However, it is challenging for traditional interdigitated electrodes (IDTs) based LWR filters to meet the requirement of a clean frequency spectrum response and enough out-of-band (*OoB*) rejection. To solve the problem, we propose LWRs with checker-shaped IDTs for the design of filters that meet the Wi-Fi 6E standard. By taking advantage of checker-shaped IDTs with unparalleled boundaries, the fabricated 6-GHz resonators successfully suppress higher-order A1 spurious modes, demonstrating a spurious-free impedance response and a high figure-of-merit (*FOM*) up to 104. Based on the demonstrated checker-shaped electrode design, the filter features a center frequency (*f*_*0*_) of more than 6 GHz, a 3 dB *BW* exceeding 620 MHz, and an excellent *OoB* rejection >25 dB, consistent with the acoustic-electric-electromagnetic (EM) multi-physics simulations. Furthermore, through the capacitance-inductance matching network technology, the filter’s voltage standing wave ratio (VSWR) is successfully reduced below 2, showing an excellent 50 Ω impedance matching. This study lays a foundation for ultra-high-frequency and ultra-wideband filters for the Wi-Fi 6/6E application.

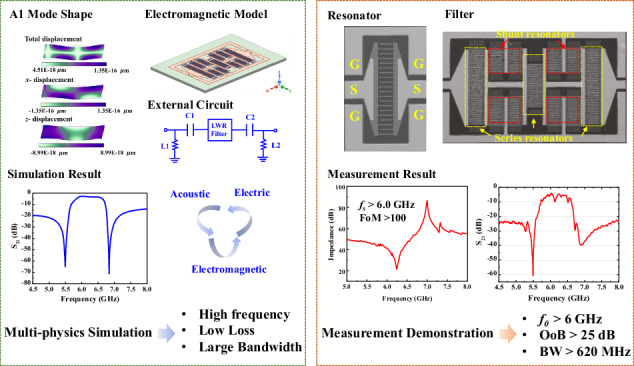

## Introduction

New services such as wireless mobile communications, the Internet of Things, and artificial intelligence, require massive and super-fast data delivery and great mobility, which necessitates that commercial filters should be operated in higher frequency, wider bandwidth, and lower losses^1,2^. Currently, popular devices such as surface acoustic wave (SAW) devices, and thin film acoustic wave resonator (FBAR) devices are facing bottlenecks in extending the frequency and bandwidth. In terms of SAW devices, the operating frequency depends on the period of the interdigitated electrode (IDT). Even though the latest technologies for Incredibly High-Performance SAW (IHP-SAW)^3^ and longitudinal leaky SAW device^4^ have been demonstrated to obtain frequencies of nearly 5 GHz, obtaining higher frequency devices requires IDTs with a narrow pitch of less than 600 nm^4,5^, which is difficult to achieve with traditional lithography equipment. For FBAR devices, currently commercially available FBAR filters rely on sputtered polycrystalline AlN thin films^6^ and can provide a high frequency of 10 GHz and a low loss, which is preferred in higher frequency applications. However, the moderate effective electromechanical coefficient (${k}_{{eff}}^{2}$ ~ 7%) do not support the filter design that requires more than 500 MHz bandwidth in the applications of 5 G new radio and Wi-Fi 6/6E. The scandium (Sc) -doped strategy is adopted to improve the piezoelectric coefficients of AlN. It is determined that the piezoelectric coefficients *d*_*33*_ of 43% Sc-doped AlN can be enhanced to 5 times than undoped AlN^7–9^. However, scandium doping leads to larger dielectric loss in the piezoelectric thin film, which lowers the quality factor (*Q*) of resonators, thus resulting in degradation of insertion loss (*IL*) and out-of-band (*OoB*) rejection in FBAR filters^6^. As a result, a resonator with both a high operating frequency and large coupling coefficients is in great demand for the next-generation RF filters.

Lamb wave resonators (LWRs) employing the first asymmetric mode (A1) based on LiNbO_3_ can support passband filtering of over 10% fractional bandwidth and the center frequency (*f*_*0*_) of more than 5 GHz based on the standard optical lithography process. This shows great potential in the application of next-generation communications^10–13^. Meanwhile, the operating frequency of A1 mode LWR is determined by the thickness of the piezoelectric layer and the distance of the adjacent electrodes, enabling monolithic multi-band solutions without additional fabrication processes. In 2017, the LWR based on Z-cut LiNbO_3_ featuring an operating frequency of 5 GHz and a ${k}_{{eff}}^{2}$ of 29%, was proposed by Yang et al^14^. Subsequently, Plessky et al. demonstrated a ZY-cut LiNbO_3_-based A1 LWRs with a high frequency of 4.8 GHz and a large ${k}_{{eff}}^{2}$ of 28%^13^. In 2022, Reinhardt et al. presented frequency-tunable LiNbO_3_-based A1 LWRs by changing the pitch of IDTs^5^. However, a lot of spurious modes are easily induced around the passband due to the large piezoelectric coefficients of LiNbO_3_, which severely affects the performance of filters. Several approaches have been used to suppress spurious modes. For example, Yandrapalli et al. systematically investigated the effects of the duty factor of electrodes, the thickness of LiNbO_3_ thin film, and the material properties on spurious modes^15^. A method of etching the piezoelectric layer in the gap region of IDTs is proposed to reduce the dispersion mismatch between the electrode-covered and the non-electrode-covered regions, to reduce the spurious modes^16^. The piston electrode structure is also a popular way to suppress spurious modes^17–19^. In our previous work, we propose LWRs with checker-shaped electrode structures and find this structure can efficiently improve ${k}_{{eff}}^{2}$, suppress spurious modes, and improve performance^20,21^.

In this work, an A1-mode LWR with checker-shaped IDTs based on Z-cut LiNbO_3_ thin film for a Wi-Fi 6/6E band filter is proposed. By taking advantage of checker-shaped IDTs with non-parallel boundaries, spurious modes are efficiently suppressed, resulting in a dramatic performance improvement, especially the figure of merit (*FOM* = ${k}_{{eff}}^{2}\times Q$). The performance parameters of LWRs in the state of the art are listed in Table 1. LWRs with checker-shaped IDTs are subsequently employed to form filters. Measurements show that filters with checker-shaped IDTs have a much cleaner spectrum than the filter with traditional IDTs. The filter exhibits a *f*_*0*_ of 6.17 GHz, a 3 dB bandwidth (*BW*) of 621 MHz (fractional bandwidth, *FBW* = 10%), and an *OoB* rejection of up to 25 dB, which agrees well with the acoustic-electric-electromagnetic (EM) multi-physics simulations. To further improve filter performance, a capacitor-inductance matching network is adopted and it successfully optimizes the voltage standing wave ratio (VSWR) below 2 indicating a good impedance matching. This spurious-suppressed filter design opens the possibility of A1-mode LWRs for filter synthesis in the 6 GHz wide-band applications.

**Table 1:** Comparison with different research on performances of A1-Mode LWRS

Ref.	Piezoelectric Material	*fs* (GHz)	${k}_{{eff}}^{2}$	*Q*	*FOM*
^14^	Z-cut LN	5.25	26.6%	112	29.8
^35^	Z-cut LN	5.44	26.3	70	18.4
^36^	ZY-cut LN	5.0	26%	340	88.4
^37^	Z-cut LN	5.0	20.5%	488	100.2
^24^	Z-cut LN	6.724	21.80%	330.8	72.1
This work	Z-cut LN	6.24	26.64%	389	103.6

## Analysis and design

### Target mode excitation

**Fig. 1:**
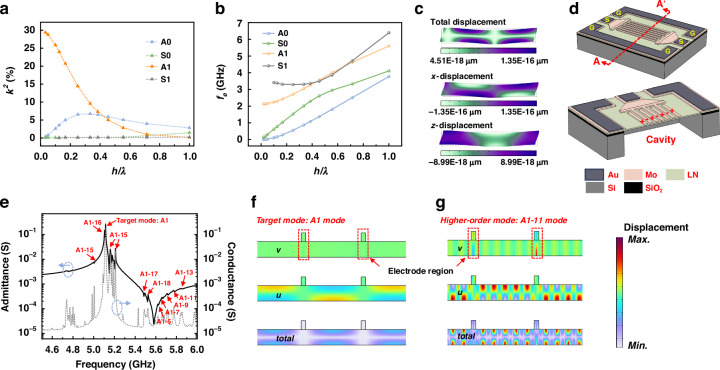
A1 mode lamb wave mode characteristics and the A1-mode LWR. **a** Calculated ${k}^{2}$ dispersions of the first four lamb wave modes. **b** Calculated eigenfrequency dispersions of the first four lamb wave modes. **c** The mode shape of the A1 mode. **d** Schematics of A1-mode LWRs. **e** Simulated Admittance and conductance curves of the LWRs equipped with traditional IDTs. **f** Simulated total displacement of the A1 mode. **g** Simulated total displacement of the A1–11 mode

The coupling coefficient (${k}^{2}$) dispersion characteristics of the first four modes of lamb waves, which propagate in the Z-cut LiNbO_3_ piezoelectric membrane (*h*_*LN*_ = 1 $\mu m$) are calculated by the finite-element method (FEM) and shown in Fig. 1a. In the FEM simulation, the model is a 2-D rectangle of the LiNbO_3_ thin film, with periodic boundary conditions on both sides in the *y-*direction. The electrodes are simplified as an infinitely thin layer and no material is defined. The ${k}^{2}$ is derived using the equation as follow^22,23^,

1 $${k}^{2}=\frac{{v}_{0}^{2}-{v}_{m}^{2}}{{v}_{0}^{2}}$$

where ${v}_{0}$ and ${v}_{m}$ are the phase velocities for a free and metalized surface of LiNbO_3_ thin film in the FEM model, respectively.

As shown in Fig. 1a, b, when *h*_*LN*_/$\lambda < \,$0.4, the A1 mode presents large ${k}^{2}$ and eigenfrequency, especially when *h*_*LN*_/$\lambda < \,$0.1, the ${k}^{2}$ of the A1 mode is more than 10 times that of other fundamental lamb wave modes. It is therefore suitable for the application of large-bandwidth and high-frequency filters. The mode shape is shown in Fig. 1c, it is obvious that the A1 mode is a thickness shear mode, and the shear deformation in the *x-*direction plays a dominant role in the total displacement. Therefore, the A1 mode can be better excited by the IDTs with a larger pitch of LWRs. The schematics of A1 mode LWR as illustrated in Fig. 1d, consist of a substrate of Si, a layer of SiO_2_, a piezoelectric layer of LiNbO_3_, an electrode layer of Mo, and a layer of Au to reduce the ohmic loss. A backside cavity is formed to confine the energy in the active region by etching the substrate. The electrode layer is patterned to two sets of IDTs. When an RF signal is applied to IDTs, A1 mode vibration will be excited by the horizontal electric field in the piezoelectric film.

The admittance and conductance responses of the LWR with traditional IDTs are depicted in Fig. 1e, and it is clear that the target mode is heavily riddled with irregularities caused by spurious modes due to internal reflections from the mechanical interfaces^16^. Standing waves can be formed only when the spurious modes and the main mode meet condition^24,25^,

2 $$p=\frac{n}{2}{\lambda }_{x}$$

and

3 $$t=\frac{n}{2}{\lambda }_{z}$$

where *p* and *t* are defined as the pitch of IDTs and the thickness of the piezoelectric layer, ${\lambda }_{x}$ and ${\lambda }_{y}$ are the wavelengths in *x-* and *z-*directions. The resonant frequency, ${f}_{s}^{{mn}}$, of the (*m*, *n*) mode can be calculated by,

4 $${f}_{s}^{{mn}}=\sqrt{{\left(\frac{m{v}_{z}}{2t}\right)}^{2}+{\left(\frac{n{v}_{x}}{2p}\right)}^{2}}$$

where ${v}_{z}$ and ${v}_{x}$ are the acoustic velocities in the *z-* and *x-*directions, *m* and *n* are mode orders in the *z-* and *x-*directions, which are equal to 1, 2, 3, etc.

As shown in Fig. 1e, the spurious modes are higher-order A1 (A1-X) modes^24^, while the higher-order fundamental asymmetric (A0) and symmetric (S0) modes are hardly to be observed, because of their smaller ${k}^{2}$ compared to the A1 mode^26^ (Fig. 1a). As a result, in this paper, we focus on the A1-X modes suppression. The total displacement of the A1 mode and the A1-X mode (taking the A1-11 mode as an example), are shown in Fig. 1f, g, respectively. It is worth noting that for the A1 mode, the vibration is concentrated in the gap region between electrodes while for the A1-11 mode, there are significant vibrations in both electrodes and the gap regions.

### Spurious modes suppression

**Fig. 2:**
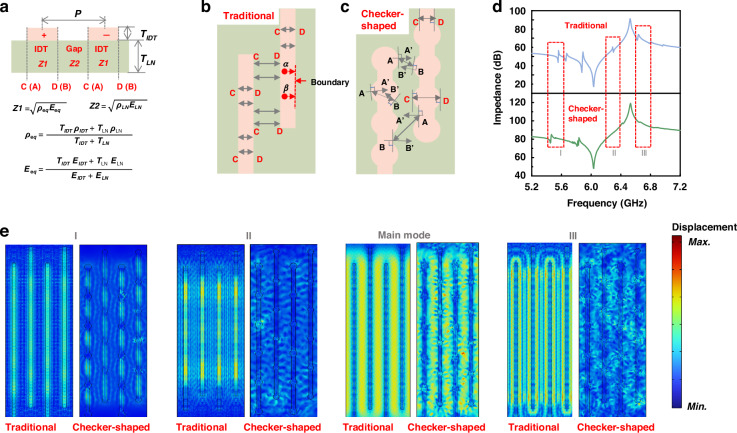
The comparisons and analysis between the traditional and checker-shaped LWRs. **a** Schematics of LWRs cross-sectional view (perpendicular to IDTs). **b** Schematics of LWRs with traditional IDTs. **c** Schematics of LWRs with checker-shaped IDTs. **d** Simulated impedance responses of LWRs with traditional and checker-shaped IDTs. **e** Simulated the total displacements of spurious modes and the A1 mode

When RF signals are applied to IDTs, a wavefront in an electrode starts propagating from an arbitrary point, $\alpha$, along the direction of the waveguide (*x*-direction). When it reaches the point, C (D) located in the boundary of the electrode, it will be reflected due to the acoustic impedance mismatch between the electrode (Z1) and the gap region (Z2) ($Z=\sqrt{\rho E}$, $\rho$ is the density, and *E* is Young’s module)^27–29^, as shown in Fig. 2a. At the same time, another wavefront starts propagating from the point of $\beta$ until it reaches at boundaries. In the case of an LWR with traditional IDTs (Fig. 2b), the two acoustic waves are reflected at the parallel boundaries at the same time, resulting in wave interference and giving rise to spurious modes^30^.

To avoid acoustic wave interference and suppress spurious modes, the structure of checker-shaped IDTs with non-parallel edges is proposed (Fig. 2c). Assuming two wavefronts propagate in the plane, one of which propagates toward point A and the other propagates toward point B (Fig. 2c). When they reach *A* and *B* respectively, they will be reflected and then travel along the antiparallel *AA’* and *BB’* directions (the tangent directions of the circle passing through points *A* and *B*). *AA’* and *BB’* are not parallel, hence, the two acoustic waves cannot interface with each other. However, when points of *A* and *B* are located on the diameter of the circle, *AA’* and *BB’* are parallel, as shown by *C* and *D* in Fig. 2c. Compared with the structure of traditional IDTs, the parallel boundaries of checker-shaped IDTs are much fewer, so the generation of spurious modes can be efficiently suppressed.

Figure 2d presents the impedance responses of LWRs with traditional and checker-shaped IDTs. Spurious modes are significantly suppressed in the LWR with checker-shaped IDTs, which is consistent with the theoretical analysis. The total displacement of the resonant of I, II, III, and the target mode in the impedance responses are shown in Fig. 2e. In the LWR with traditional IDTs, the vibration of the piezoelectric layer under the electrode region is strong. While in the LWR with checker-shaped IDTs, the displacement in the electrode is weakened and the distribution of the vibration is scattered because of the destruction of the coherent formation of the standing waves by antiparallel electrode edges.

**Fig. 3:**
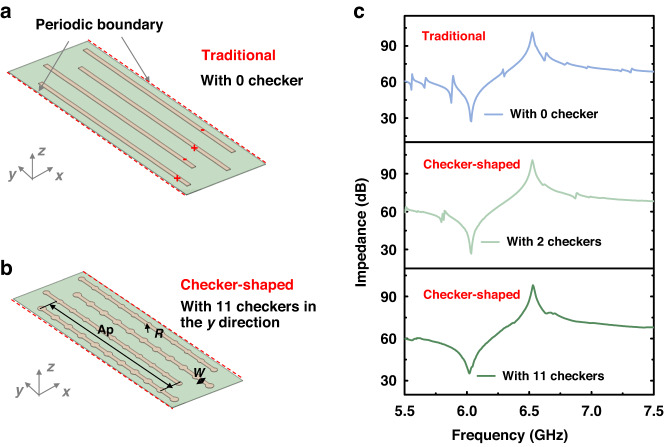
FEM models and results. **a** The FEM model of LWRs with traditional IDT. **b** The FEM model of LWRs with checker-shaped IDTs. **c** Simulated results of impedance responses of LWRs with different structures of IDTs

Furthermore, as the number of checkers increases, the parallelism of the electrode edges decreases correspondingly. Therefore, based on the same resonator Ap, the relationship between the efficiency of spurious mode suppression and the degree of the anti-parallel edge is studied by increasing the number of checkers per electrode. Figure 3a, b shows FEM models of the LWRs with traditional and checker-shaped IDTs, and the Ap is 80 μm. Both sides are applied with periodic boundaries to eliminate the effect of the reflection caused by free edges^31,32^. Simulation results of impedance curves are shown in Fig. 3c. As the number of checkers increases from 0 to 11, spurious modes are gradually suppressed, especially in the band to the left of anti-resonant frequency (*f*_*p*_).

**Fig. 4:**
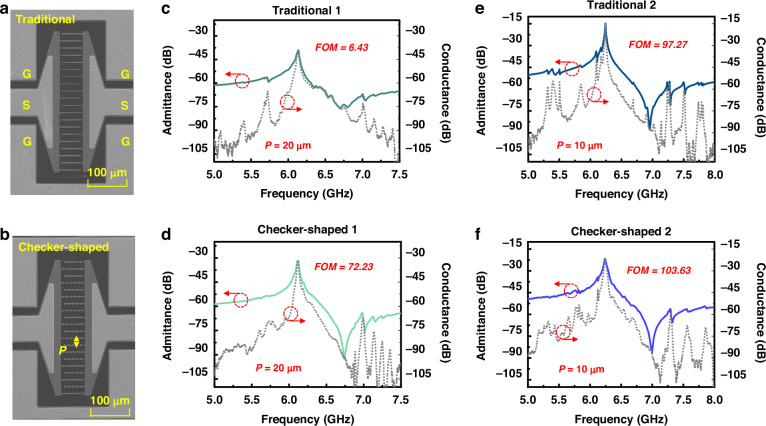
Experimental results of fabricated LWRs. **a** The SEM image of LWRs with traditional IDTs. **b** The SEM image of LWRs with checker-shaped IDTs. **c** Measured admittance and conductance curves of LWRs with traditional IDTs and a *P* of 20 ${\rm{\mu }}{\rm{m}}$. **d** Measured admittance and conductance curves of LWRs with checker-shaped IDTs and a *P* of 20 ${\rm{\mu }}{\rm{m}}$. **e** Measured admittance and conductance curves of LWRs with traditional IDTs and a *P* of 10 ${\rm{\mu }}{\rm{m}}$. **f** Measured admittance and conductance curves of LWRs with checker-shaped IDTs and a *P* of 10 ${\rm{\mu }}{\rm{m}}$

To demonstrate the idea, LWRs with traditional and checker-shaped IDTs are fabricated, as shown in Fig. 4a, b, respectively. The piezoelectric layer is a 300 nm thick Z-cut LiNbO_3_ thin film, the electrode is Mo with a thickness of 300 nm, and a layer of Au is formed on the surface of Mo to reduce ohmic loss. Prepared LWRs can be divided into two groups, one with a *P* of 20 ${\rm{\mu }}{\rm{m}}$ and the other with a *P* of 10 ${\rm{\mu }}{\rm{m}}$. All of the structure parameters are listed in Table 2. The measured results of LWRs are in agreement with simulations. Figure 4c, d are measured admittance and conductance responses of LWRs with a *P* of 20 ${\rm{\mu }}{\rm{m}}$, showing a resonant frequency (*f*_*s*_) of around 6 GHz and a ${k}_{{eff}}^{2}$ up to 20% (${k}_{{eff}}^{2}=\frac{{\pi }^{2}}{4}\frac{\left({f}_{p}-{f}_{s}\right)}{{f}_{p}}$). For LWRs with a *P* of 20 μm, the admittance response of the LWR with traditional IDTs, a lot of spurious modes appear, especially near the *f*_*p*_, frequency, which severely deteriorates the performance of the resonator, causing a non-ideal impedance ratio, *Z*_*ratio*_, of 36.89 dB (*Z*_*ratio*_ = *Z*_*p*_–*Z*_*s*_, *Z*_*s*_ and *Z*_*p*_ represent the impedance at the resonant and anti-resonant frequencies), and a low *FOM* of 6.43. On the contrary, the impedance response of the LWR with checker-shaped IDTs shows a much smoother spectrum, resulting in a *Z*_*ratio*_ of 60.76 dB and an excellent *FOM* of 72.23, in which *FOM* is more than 10 times higher than the LWR using traditional IDTs. As for LWRs with a *P* of 10 μm, the *FOM* of the LWRs with traditional and checker-shaped IDTs are 97.27 and 104, and the *Z*_*ratio*_ are 73.27 dB and 64.82 dB. Although the *Z*_*ratio*_ is slightly lower than that of the LWR with the traditional electrode due to the scattering of the acoustic wave by the unparallel boundary, the higher-order A1-mode spurious modes are suppressed and the *FOM* is improved, which is essential for fabricating a filter with a flat passband and low loss for the application of Wi-Fi 6/6E. The comparison of the performances of the fabricated A1-mode LWR in this work and previous research are listed in Table 1.

**Table 2:** Design parameters of the fabricated A1-mode LWRS

Resonator	*T*_*LN*_ (nm)	*T*_*IDT*_ (nm)	*P* (μm)	*Ap* (μm)	*W* (μm)	*N* (pair)	*N_C*	*R* (μm)
Traditional 1	300	300	20	55	4	10	0	0
Checker-shaped 1	300	300	20	55	/	10	6	2
Traditional 2	300	300	10	55	2	10	0	0
Checker-shaped 2	300	300	10	55	/	10	6	2

### LWR filter design

**Fig. 5:**
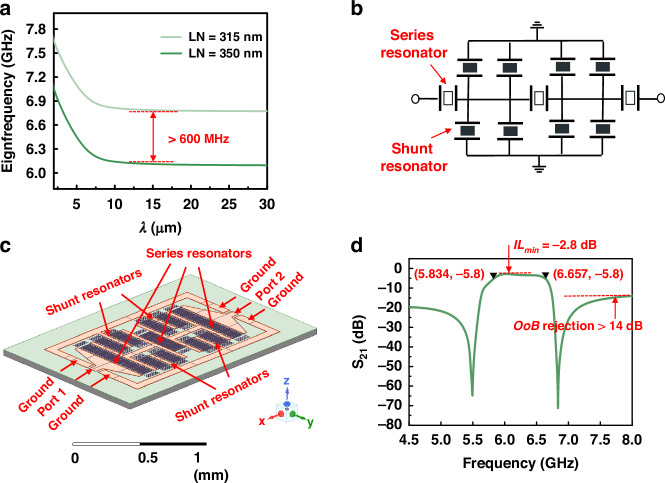
The design and simulation results of the A1-mode LWR filter. **a** Eigenfrequency dispersion curve of the A1 mode in 350 nm and 315 nm Z-cut LiNbO_3_ thin films. **b** Schematic circuit design of the LWR filter. **c** EM simulation model of A1-mode LWR filter. **d** *S*_*21*_ response of the multi-physics simulation

With the optimized A1-mode LWRs with checker-shaped IDTs, a ladder topology consisting of series and shunt resonators is adopted for the ultra-high-frequency and ultra-wideband LWR filter demonstration. To achieve a filter with a *f*_*0*_ of 6.2 GHz and an *FBW* of more than 10%, the thicknesses of Z-cut LiNbO_3_ thin films in series and shunt resonators are chosen to 300 nm and 315 nm, respectively. Figure 5a shows the eigenfrequency dispersion characteristics of A1 mode acoustic wave propagating in the Z-cut LiNbO_3_ thin films with thicknesses of 300 nm and 315 nm. Evidently, the A1-mode wave exhibits strong dispersion especially when $\lambda$ ($\lambda$ = 2*P) is smaller than 15 ${\rm{\mu }}{\rm{m}}$. As a result, the $\lambda$ is chosen to be 20 $\mu m$ to easily obtain the right frequency of resonators and enough frequency shift (>600 MHz). The schematic circuit of the fabricated filter is shown in Fig. 5b, and it consists of 7 elements, including 3 series resonators and 4 sets of shunt resonators. To make the filter footprint compact and symmetric, shunt resonators are split into two identical resonators distributing evenly on both sides of the series resonators. Table 3 lists the design parameters of series and shunt resonator arrays, where *T*_*IDT*_*, T*_*LN*_, and *P* are defined in Fig. 2a, Ap, *W*, and *R* are defined in Fig. 3a. *N* represents the pair of interdigital electrodes.

**Table 3:** Design parameters of the series and shunt resonators of LWR filters

Resonator	*T*_*LN*_ (nm)	*T*_*IDT*_ (nm)	*P* (μm)	*Ap* (μm)	*W* (μm)	*N* (pairs)	*C*_*0*_ (fF)
Series	315	300	10	90	5	35	490
Shunt	350	300	10	90	5	12	150

Electromagnetic (EM) simulations are used to further evaluate the performance of filters, such as the *IL*_*min*_ and the *OoB* rejection, where the lead loss and electromagnetic coupling caused by filter interconnection and layout are taken into consideration^33^. The EM simulation model and the *S*_*21*_ response of filters based on the multi-physics simulation are shown in Fig. 5c, d. The *S*_*21*_ response shows that the *f*_*0*_ is 6.23 GHz, the minimum insertion loss (*IL*_*min*_) is −2.8 dB, and the *OoB* rejection is greater than 14 dB.

### Fabrication process

**Fig. 6:**
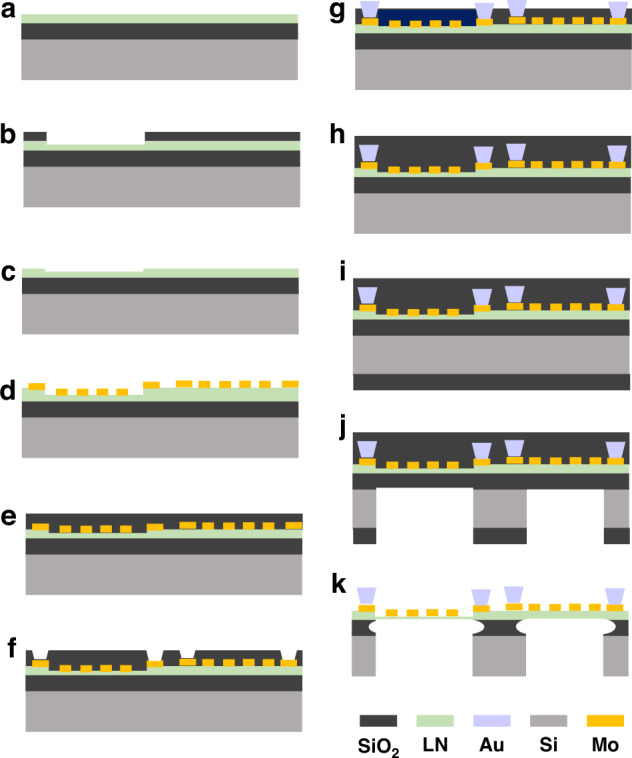
Fabrication process steps for LiNbO_3_-based A1-mode LWR filters. Fabrication process of the A1-mode LWR filter based on the Z-cut LiNbO_3_ thin film. **a** LiNbO_3_ wafer. **b** SiO_2_ layer is deposited and etched. **c** LiNbO_3_ is partially etched to obtain the frequency shift between the series and shunt resonators. **d** The Mo layer is deposited and patterned. **e** SiO_2_ layer is prepared for the lift-off process of Au. **f** SiO_2_ layer is etched. **g** The Au layer is formed to reduce ohmic loss. **h** SiO_2_ layer as a protective layer is formed. **i** The SiO_2_ layer on the back-side wafer is deposited as a hard mask for the Si etching. **j** The substrate is etched to define the device. **k** The device is released by a wet etching process

The designed filters are fabricated following the process shown in Fig. 6. Filters are implemented on 350 nm thick Z-cut LiNbO_3_ thin film (Fig. 6a, Jinan Jingzheng Electronics Co., Ltd.), which is obtained by a layer transfer process. A layer of 200 nm SiO_2_ is deposited as a hard mask to protect LiNbO_3_ of shunt resonators from etching by a process of plasma-enhanced chemical vapor deposition (PECVD) and etched by a process of reactive ion etching (RIE). Subsequently, partial etch LiNbO_3_ in the area where series resonators sit to 315 nm by a process of ion beam etching (IBE) to obtain the frequency shift between the shunt and series resonators. After LiNbO_3_ thin film etching, SiO_2_ is stripped by a wet etching process. Then, a layer of Mo is sputtered and patterned as IDTs. A layer of SiO_2_ is deposited by a process of PECVD for the lift-off process of a layer of 500 nm Au. After the layer Au is defined, an additional SiO_2_ layer is formed on the back side of the wafer as a hard mask to etch the Si substrate by a process of deep reactive ion etching. Finally, the device is released by 7:1 Buffered Oxide Etch (BOE)-based wet etching.

## Result and discussion

**Fig. 7:**
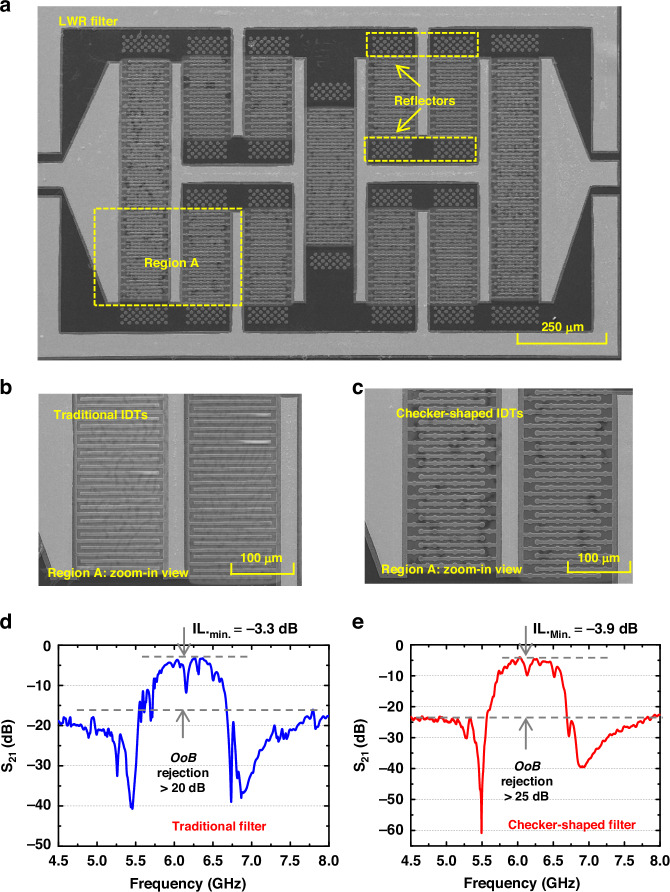
Experimental results of fabricated A1-mode LWR filters. **a** SEM images of the fabricated LWR filters. **b** A zoom-in view of region A in the filter with traditional IDTs. **c** A zoom-in view of region A in the filter with checker-shaped IDTs. **d** The measured *S*_*21*_ responses of the LWR filter with traditional IDTs. **e** The measured S21 responses of the LWR filter with checker-shaped IDTs

Figure 7a–c shows the SEM images of the fabricated A1-mode LWR filter, a close-up view of the region A with traditional and checker-shaped IDTs, respectively. Measured results of transmission responses of LWR filers with traditional and checker-shaped IDTs are displayed in Fig. 7d, e, respectively. To further reduce the energy loss, improve the *Q* of the resonator, and reduce the *IL* of the filter, two sets of open-circuit acoustic reflectors are used^34^. The acoustic reflectors are comprised of checker-shaped acoustic reflector arrays with a distance of half of the wavelength formed in the direction of acoustic wave propagation to reflect the leaked acoustic wave to the resonator.

**Fig. 8:**
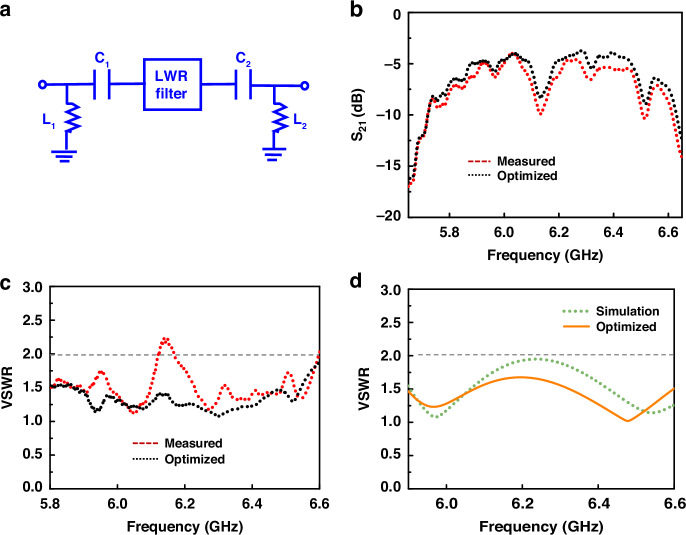
Filter optimization. **a** External optimization circuit model. **b** Comparison *S*_*21*_ responses between the measurement and after adding an external optimization result. **c** Comparison of VSWR responses of the fabricated filter before and after adding an external optimization circuit. **d** Comparison of VSWR responses of the simulated filter before and after adding an external optimization circuit

Compared with the *S*_*21*_ response of the filter with traditional IDTs, the filter with the checker-shaped electrode design shows notable suppression of spurious modes with both roll-off and in-band ripples expressively enhanced, demonstrating the effectiveness of the resonator design. Furthermore, the fabricated filter with checker-shaped IDTs shows a *f*_*0*_ of 6.17 GHz, a 3 dB *BW* of 621 MHz (*FBW* = 10%), an *IL*_*min*_ of −3.9 dB, and a large *OoB* rejection > 25 dB, which has an excellent agreement with the result of EM simulation (Fig. 8b).

The capacitor-inductance matching network is adopted to optimize the in-band ripple and further improve the performance of the filter. The external circuit diagram and related parameters are shown in Fig. 8a and Table 4, respectively. Figure 8b shows the *S*_*21*_ responses of the measured filter results between before and after optimization. The in-band ripple has been improved. Besides, it can be found that the voltage standing wave ratio (VSWR) is reduced to less than 2 in the passband after adding the capacitor-inductance matching network (Fig. 8c), which means that less energy is reflected and more signal can reach the output. Moreover, the filter in the simulation is also optimized by the external circuit. The result can be seen in Fig. 8d. The VSWR is reduced from 1.95 to 1.65, significantly improving 50 Ω impedance matching.

**Table 4:** Parameters of the capacitor-inductance matching network

	*C*_*1*_ (pF)	*C*_*2*_ (pF)	*L*_*1*_ (nH)	*L*_*2*_ (nH)
For the Result of Measurement	5	1.79	4.10	1.56
For the Result of EM simulation	8.622	1.07	4.70	3.08

## Conclusion

In this paper, filters based on A1-mode LWRs with a center frequency of 6.17 GHz using Z-cut LiNbO_3_ thin films are investigated. LWRs with checker-shaped IDTs are proposed, which is demonstrated excellent spurious mode suppression by theoretical and experiment results. The fabricated LWR simultaneously achieves a spurious-suppression response, a high operating frequency greater than 6 GHz, and an excellent *FOM* of 104, which is essential for a high-performance filter with low loss and sufficient signal isolation in and out of the band. According to the result of the acoustic-electric-electromagnetics multi-physics simulation, the designed filter exhibits an *IL*_*min*_ of less than 2.8 dB, a bandwidth of more than 820 MHz, and an *OoB* rejection of more than 15 dB. Then, the filter is prepared, and the measured results present that the filter with checker-shaped IDTs shows a much cleaner *S*_*21*_ response than that of the filter based on traditional IDTs and has a *f*_*0*_ of 6.17 GHz, a 3 dB *BW* of 621 dB, the *OoB* rejection > 20 dB, and an *IL*_*min*_ of −3.9 dB, which agrees well with simulations. To further improved the filter’s performance, an external capacitor-inductance matching circuit is adopted and the VSWR is successfully suppressed below 2, indicating the filter has an excellent 50 Ω impedance match and showing a strong potential for next-generation RF front-end applications.
